# Chronic consumption of a western diet induces robust glial activation in aging mice and in a mouse model of Alzheimer’s disease

**DOI:** 10.1038/srep21568

**Published:** 2016-02-18

**Authors:** Leah C. Graham, Jeffrey M. Harder, Ileana Soto, Wilhelmine N. de Vries, Simon W. M. John, Gareth R. Howell

**Affiliations:** 1 The Jackson Laboratory, 600 Main St, Bar Harbor, ME, USA; 2 Graduate Program in Genetics, Sackler School of Graduate Biomedical Sciences, Tufts University, 136 Harrison Avenue, Boston, MA 02111, USA; 3 Howard Hughes Medical Institute, 600 Main St, Bar Harbor, ME, USA; 4 Department of Ophthalmology, Tufts University School of Medicine, Boston, MA, USA

## Abstract

Studies have assessed individual components of a western diet, but no study has assessed the long-term, cumulative effects of a western diet on aging and Alzheimer’s disease (AD). Therefore, we have formulated the first western-style diet that mimics the fat, carbohydrate, protein, vitamin and mineral levels of western diets. This diet was fed to aging C57BL/6J (B6) mice to identify phenotypes that may increase susceptibility to AD, and to APP/PS1 mice, a mouse model of AD, to determine the effects of the diet in AD. Astrocytosis and microglia/monocyte activation were dramatically increased in response to diet and was further increased in APP/PS1 mice fed the western diet. This increase in glial responses was associated with increased plaque burden in the hippocampus. Interestingly, given recent studies highlighting the importance of TREM2 in microglia/monocytes in AD susceptibility and progression, B6 and APP/PS1 mice fed the western diet showed significant increases TREM2+ microglia/monocytes. Therefore, an increase in TREM2+ microglia/monocytes may underlie the increased risk from a western diet to age-related neurodegenerative diseases such as Alzheimer’s disease. This study lays the foundation to fully investigate the impact of a western diet on glial responses in aging and Alzheimer’s disease.

Alzheimer’s disease (AD) is an age-related neurodegenerative disease characterized by amyloid-beta plaques (Aβ), neuronal dysfunction and neuroinflammation^1^,^2^. There are two types of AD, early onset AD (EOAD) and late onset AD (LOAD). EOAD is commonly caused by mutations in genes for amyloid precursor protein (APP) and presenilins 1 and 2 (PSEN1 and PSEN2)^3^,^4^. In contrast, LOAD is a complex, age-related disease and little is known about how certain risk factors contribute to LOAD susceptibility and pathogenesis. For example, genome-wide association studies (GWAS) have identified variations in apolipoprotein E (*APOE*), triggering receptor expressed on myeloid cells 2 (*TREM2*) and complement receptor 1 (*CR1*) as risk factors for AD, but the direct connections of these genes with AD are still not clear^5^,^6^,^7^. Lifestyle choices such as diet and exercise are also critical factors in the development of AD. A lack of exercise is reported to be a contributing factor in over 20% of AD cases in the US^8^,^9^. It has also been suggested that certain diets or diet-induced obesity can increase or decrease the chances for cognitive decline and AD^8^,^9^,^10^. Therefore, a better understanding of how lifestyle choices, particularly diet, increase susceptibility to and progression of AD is required.

The effects of an increasing population eating western diets while doing little activity on a daily basis are leading to a decline in health and an increase in the diagnosis of chronic diseases^11^. Diseases that are influenced by diet, such as AD, are the greatest cause of morbidity and mortality in the western world including the United States. A western diet tends to include highly processed, less expensive fast food^12^,^13^, that has a high fat content and simple carbohydrates while lacking essential nutrients from complex grains, fruits and vegetables^10^,^11^,^14^,^15^,^16^. Although some work has focused on the effects of specific components of a western diet, for instance, high fat and cholesterol, on plaque accumulation and cognitive function^17^,^18^,^19^,^20^,^21^ the overall impact of a western style diet on AD has not been studied. The strength of the western diet we created for this study is that it incorporates many aspects of a western diet. Previous studies showing increased plaque load and cognitive dysfunction in AD transgenic mice are in response to diets with 40–60% saturated fat, which is not accurate in mimicking a true western diet^17^,^20^,^21^,^22^. Therefore, despite studies suggesting a link between a western diet and dementias, such as AD^23^, the mechanisms by which a western diet increases risk for AD are not known.

A western diet can cause nutrient deficiency and inflammation that could impact cognition directly. However, a western diet in combination with physical inactivity can lead to obesity that increases risk of cognitive decline and AD. Currently, more than 35% of Americans over the age of 65, and 40% of middle-aged (40–59 years old) individuals, are obese^24^. Some studies suggest obesity, particularly mid-life obesity, increases the chances of cognitive decline and AD by six-fold^8^,^9^. Increased immune response, such as inflammation, is one of the major consequences of a western diet and/or obesity. Multiple studies suggest that increased cerebral innate immune responses (or neuroinflammation) are correlated with memory loss in patients with various diseases, including AD and traumatic brain injury^25^,^26^. In mice, consumption of a high fat diet caused neuroinflammation and cognitive decline^17^,^19^,^22^. These results suggest that diet-induced immune responses will affect susceptibility to or progression of AD, but specific immune responses that may be damaging, as apposed to beneficial, have not been determined.

Recent data suggest that innate immune responses by myeloid cells such as resident microglia and infiltration of blood-derived macrophages likely represent an important link between a western diet, cognitive decline and AD. For instance, obesity-induced inflammation is reported to result in an increase of myeloid cells in many tissues, including in the brain^27^,^28^. Myeloid cells (including resident microglia and infiltrating macrophages monocytes) expressing high levels of *TREM2* contribute to cognitive decline and dementia^29^,^30^,^31^. TREM2+ myeloid cells are also present surrounding plaques in mouse models of AD^29^,^32^. The presence of these TREM2+ cells has been reported to amplify neuroinflammatory cytokines and plaque load in AD mouse models^29^,^33^. However, whether TREM2+ cells are impacted by a western diet in the absence of genetic risk factors for AD has not been studied.

Given that multiple epidemiological studies implicate western diet as a risk factor for AD susceptibility and progression, we formulated a chow for mice that closely mimicked diets that are commonly consumed in western countries. This western diet chow was higher in calories and lower in nutrient densities compared to standard mouse diets and contained animal-based rather than plant-based proteins and fats. This is the first time the cumulative effects of the dietary factors that constitute a western diet have been studied in brains of mice. Further, given that other studies have tended to assess the short-term effects of dietary factors, we determined the long-term effects of western diet consumption on AD susceptibility and progression by feeding it to C57BL/6J (B6) and APP/PS1 mice for 8 months (from 2 to 10 months of age). We found that long-term western diet consumption caused a dramatic increase in cerebral innate immune responses by astrocytes and myeloid cells in both B6 and APP/PS1 mutant mice. The western diet increased potentially damaging glial cells in B6 mice. It also increased the number of TREM2+ myeloid cells surrounding plaques that correlated with more plaque deposition in APP/PS1 mice.

## Methods

### Mouse strains and husbandry

B6.Cg-Tg(APPswe,PSEN1dE9)85Dbo/Mmjax mice (MMRRC stock #034832-JAX; JAX stock #5864)^34^,^35^ referred to as B6.*APB*^*Tg*^ or APB) were developed by Dr. David Borchelt, and obtained from the Mutant Mouse Resource and Research Center (MMRRC) at The Jackson Laboratory. To generate experimental cohorts, B6.*APB*^*Tg*^ mice were mated to C57BL/6J (B6, JAX stock #000664) mice to generate both B6.*APB*^*Tg*^ and wild type (referred to as B6) mice. Standard genotyping protocol for *PSEN*^*dE9*^ was followed to confirm the presence of the *APP*^*swe*^/*PSEN1*^*dE9*^ transgenes (see http://jaxmice.jax.org/strain/005864). All mice were maintained on a 12/12 hours (hrs) light/dark cycle. Mice were group-housed, dependent on sex at wean, in 6 inch duplex wean cages with pine shavings. Both males and females were used in this study. Cohorts of B6 and B6.*APB*^*Tg*^ mice were maintained from wean on standard LabDiet ^®^ 5K54 (referred to as “control diet”) and TestDiet^®^ 5W80 (Western diet, WD) adapted from TestDiet^®^ 5TLN with added high fructose corn syrup, lower fiber and increased milk protein and fat (Table 1). The Animal Care and Use Committee at The Jackson Laboratory approved all procedures used in this study. Daily monitoring of mice via routine health care checks were carried out to determine their general well being. Approximately 10% of mice fed the western diet developed dermatitis and were eliminated from this study using an IACUC approved CO_2_ euthanasia protocol.

### Mouse Phenotyping

#### Body composition

Mice were assessed at 9 months of age by dual energy X-ray absorptiometry (DEXA) using a Lunar PIXImus densitometer (GE Medical Systems) after mice were anesthetized with tribromoethanol (0.2 ml 2% solution/10 g body weight). The skull is omitted from the DEXA analysis because of its high bone density. Mice were weighed on an Ohaus Navigator scale with InCal calibration to accommodate animal movement.

#### Blood glucose

Mice were assessed at 9.5 months of age, two weeks prior to tissue harvesting, using an Abbott Laboratories AlphaTRAK Blood Glucose Monitoring System. Mice were fasted for 8 hours prior to measurements, and blood collected using a tail snip.

### Tissue harvesting, protein isolation and sectioning

At 10 months of age mice were administered a lethal dose of Ketamine/Xylazine by intraperitoneal injection, and transcardially perfused with 1xPBS (phosphate buffered saline). Brains were dissected, and the right hemisphere snap frozen for protein isolation, while the left hemisphere was fixed in 4% paraformaldehyde overnight at 4 °C. The fixed hemispheres were rinsed with 1xPBS, cryoprotected in 10% sucrose, followed by 30% sucrose at 4 °C, and finally embedded in OCT (optimal cutting temperature compound). Frozen brains were sectioned at 25 μm and stored at −80 °C until required. Protein was extracted with Trizol Reagent (Life Technologies Cat#15596-018) following manufacturer’s guidelines. Protein pellets were resuspended in a solution of 1:1 8M urea and 1% SDS.

### Immunofluorescence, Thioflavin S staining, and image capture

Cryosections were rinsed with PBT (1xPBS with 1% TritonX-100) for 5 minutes (mins) then incubated with 500 μL of Liberate Antibody Binding Solution (L.A.B. -Polysciences Inc.) solution for 20 minutes at room temperature (RT) for antigen retrieval. Slides were then incubated overnight at 4 °C in the following primary antibodies: rabbit polyclonal anti-GFAP (1:200, Dako); rabbit polyclonal anti-IBA1 (1:250, Wako); rabbit polyclonal anti-NeuN (1:100, Cell Signaling Inc); mouse monoclonal anti-non-phosphorylated neurofilament (1:200, Covance) and sheep polyclonal anti-TREM2 (1:200, RD Systems). The sheep polyclonal anti-TREM2 antibody was previously verified using Trem2 deficient mice^36^,^37^. All antibodies were diluted in PBTB (1xPBS, 1% TritonX-100 and 1%BSA) containing 10% normal goat or donkey serum. After primary incubation, sections were washed 3 times in PBT and incubated with appropriate secondary antibodies (goat anti-rabbit Alexa Fluor 488/594/633, goat anti-mouse Alexa Fluor 488, donkey anti-sheep Alexa Fluor 594, 1:1000 dilution, Life Technologies) for 2 hrs at RT. All sections were then counterstained with DAPI and mounted with Aqua PolyMount (Polysciences). For Thioflavin S staining, sections stained with IBA1 and GFAP were further counterstained with 1% Thioflavin S (diluted in a 1:1 water:ethanol ratio). Slides were incubated for 8 mins at RT in 1% Thioflavin-S, washed in 80% ethanol, then 95% ethanol and finally in dH2O and mounted. Images were taken using either the Leica SP5 confocal microscope or the Zeiss Axio Imager.Z2. For each antibody, all images were captured using identical parameters for accurate quantification.

Initial observations were performed in sections from both males and females. Quantification of cell numbers was performed on brain sections from at least 4–6 male mice, as there was no overt difference between sexes. For plaque counts, the number of plaques present in the entorhinal cortical region for each mouse was determined. For IBA1^+^ cells, 5 equally spaced images were captured (using 20× optical lens) of either the cortex, in the region of the entorhinal cortex, or the hippocampus, from a central brain section of each mouse. For NeuN^+^ cells, 5 equally spaced images were captured (using 20× optical lens). For IBA1^+^ cells associated with plaques, images of 8+ plaques per brain were imaged (using 20× optical lens). Images were processed and all cells in the 20× image were counted using the cell counter plugin for ImageJ/FIJI. A single cell was determined as a DAPI stained nucleus associated with a cell specific antibody stain (e.g. IBA1or NEUN). Cell numbers in the 5 images from each mouse were totaled and then averaged across mice. Mouse number and diet were masked to the investigator for all cell counting assays.

### ELISA

Aβ42 levels were determined using the Life Technologies detection kit (cat#KHB3442) following the specified instructions. Four male and three female B6.*APB*^*Tg*^ mouse samples were used. Protein samples were diluted 1:50 in standard diluent buffer to ensure that the levels of urea and SDS were compatible with the ELISA assay kit (cat#KHB3442). Samples were then compared to a standard curve and Aβ42 concentrations were established as per manufacturers recommendations.

### Statistical tests

Data is expressed as mean ± SEM. Statistical significance was determined by ANOVA with Bonferroni correction or Student’s two-tailed t tests, performed using GraphPad or Excel. For each test, p < 0.05 was significant. Specific p values for each test are listed in the figure legends.

## Results

### The western diet induces obesity but not diabetes in B6 mice

In general, laboratory mice are fed a nutritionally optimized diet that does not model the diet eaten by the majority of people in the western world. Therefore, to explore the impact of a western diet on age-related changes in the brain, we developed a diet for mice that mimics diets commonly consumed by western cultures^11^. Unlike other studies that have assessed one or two components of a western diet (such as high fat or high cholesterol)^17^,^18^,^19^,^20^,^21^, our goal was to develop a diet that incorporated as many of the components of a western diet as possible. The critical dietary factors that have been altered from a standard mouse chow to formulate our western diet include (i) fat content and source, (ii) carbohydrate content and source, (iii) sugar content and source and (iv) a decrease in essential vitamins (Table 1). This is the first time these four major factors of a western diet have been assessed in combination. Specifically, the western diet has elevated fat content (achieved using a combination of milk fat, vegetable shortening and lard) with added cholesterol, saturated and monounsaturated fatty acids, as well as low omega-3 fatty acids and linoleic acids (deficiency of which is thought to be detrimental)^11^,^15^,^23^. Overall, essential mineral and vitamin levels in the western diet are below the recommended dose^11^. Sucrose and high fructose corn syrup make up the sugar content in the western diet. Sugar is not added to standard mouse chow. Protein contained in the western diet is also sourced from casein, found in milk, whereas protein from the control diet is sourced from grain. As is the case in a western diet in the human population, the total protein levels are similar between our western diet and the control diet, as are energy content – approximately 4 (kcal/g)^2^ – in order to achieve a similar calorie content from the same amount of food.

**Figure 1:**
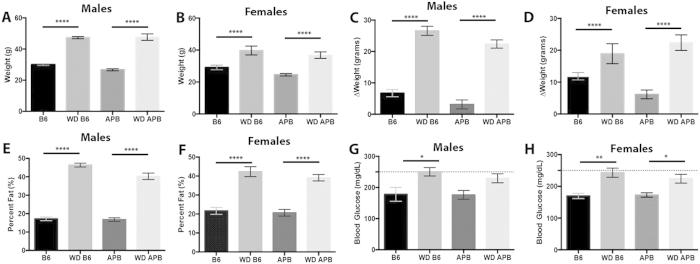
The western diet 5W80 induces obesity in mice. (**A**) Weight (grams, g) increases in male B6 and B6.*APB*^*Tg*^ after being fed a western diet (WD) for 8 months (p < 0.0001, n = 5) **(B)** Weight (g) also increases in female B6 and B6.*APB*^*Tg*^ after being fed a western diet for 8 months (p < 0.0001, n = 4). **(C)** Weight increased by approximately 20 grams in male B6 and B6.*APB*^*Tg*^ mice fed a western diet compared to mice fed the control chow (p < 0.0001, n = 5). **(D)** Weight increased by approximately 10 grams in female B6 and B6.*APB*^*Tg*^ mice fed a western diet compared to mice fed the control chow (p < 0.0001, n = 4). **(E)** Body fat percentage in WD-fed mice is significantly increased in both B6 and B6.*APB*^*Tg*^ male mice (p < 0.0001, n = 5). **(F)** Body fat percentage in WD-fed mice is significantly increased in both B6 and B6.*APB*^*Tg*^ female mice (p < 0.0001, n = 4). **(G)** Fasting blood glucose levels are increased in male B6 and B6.*APB*^*Tg*^ mice fed the western diet (p = 0.0072, n = 5)**. (H)** Fasting blood glucose levels are increased in female B6 and B6.*APB*^*Tg*^ mice fed the western diet. Dotted line in (G, H) indicates diabetic blood glucose threshold. (p = 0.001, n = 4).

To assess the effect of long-term consumption of a western diet (also referred to as WD) on AD susceptibility and progression, B6 and APP/PS1 (herein referred to as B6.*APB*^*Tg*^) mice were fed the western diet or control diet from 2 months of age (mos) and assessed at 10 mos. Modeling the effects of chronic consumption of a western diet in the human population^11^,^15^,^23^,^38^, B6 and B6.*APB*^*Tg*^ mice fed the western diet were significantly heavier than B6 and B6.*APB*^*Tg*^ mice fed the control diet. These results were observed in both male and female mice (Fig. 1A,B). Weight gain from 2 mos to 10 mos in mice fed WD is significantly greater than the average weight gain of control chow-fed mice throughout lifetime (Fig. 1C,D). Interestingly, male mice fed a western diet showed a greater increase in weight than female mice. WD-fed mice have significantly more fat than mice fed the control diet (Fig. 1E,F). Importantly, western diet-fed mice, independent of genotype, were considered obese based on body fat percentages (>30%)^39^. Previous studies state that blood glucose levels greater than 250 mg/dL are indicative of diabetes in mice^37^. Blood glucose measurements were significantly increased in western diet-fed mice independent of genotype, but remained below 250 mg/dL and therefore the mice were not considered to be diabetic (Fig. 1G,H). Therefore, under the conditions tested in this study, the western diet induced obesity in both B6 and B6.*APB*^*Tg*^ mice by 10 mos and was accompanied by a pre-diabetic elevation in blood glucose levels.

### The western diet increases neuroinflammation and causes neuronal cell loss in B6 mice

**Figure 2:**
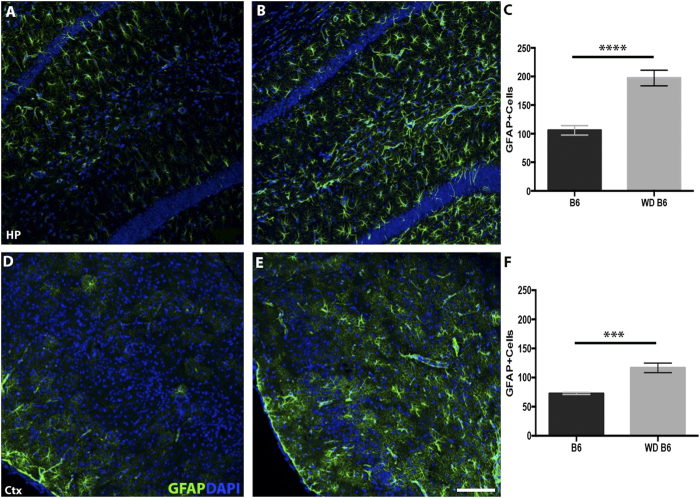
Astrocytosis increases in response to a western diet in B6 mice. (**A**,**B)** Immunofluorescent staining of glial fibrillary acidic protein (GFAP, green) in the hippocampus (HP) increases in B6 mice fed the western diet (**B**) compared to control diet (**A**). **(C)** The number of GFAP+ cells increase in the hippocampus of western diet-fed mice compared to control diet (p < 0.0001, n = 7). **(D,E)** Immunofluorescent staining of GFAP in the entorhinal cortex (Ctx) increases in B6 mice fed a western diet (**E**) compared to control diet (**D**). (**F)** GFAP+ cell number increases in the entorhinal cortex of B6 mice fed the western diet compared to control diet (p = 0.0002, n = 7). Blue  =  DAPI. Scale bars: 160 μm.

**Figure 3:**
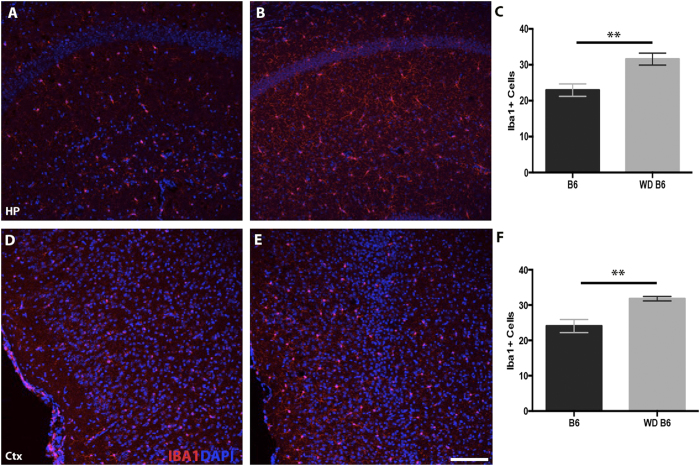
Microglia/monocytes increase in response to western diet in B6 mice. (**A**–**C**) The number of IBA1+ cells (red) increase in the hippocampus of western-diet fed B6 mice (**B**) compared to control mice (**A**) (p = 0.005, n = 6, **C**). (**D**–**F)** IBA1+ cell number also increases in the entorhinal cortex of western diet-fed B6 mice (**E**) compared to control mice (**D**) (p = 0.0043, n = 6, **F**). Blue  =  DAPI. Scale bars: 160 μm.

To assess the impact of chronic consumption of a western diet on the aging brain, brains of B6 mice fed the western diet were compared to brains of B6 mice fed the control diet. Overall, the morphology of the brain was similar between WD and control samples, with no obvious changes to cortical and hippocampal organization and thickness. However, the western diet had a major impact on glial cell activity. Brains from WD-fed mice were assessed for levels of glial fibrillary protein (GFAP), a marker of reactive astrocytosis, and allograft inflammatory factor 1 (AIF1, commonly known as IBA1), a marker of microglia/monocyte activation. We focused particularly on the hippocampus and entorhinal cortex as these regions are selectively and highly affected in AD. WD-fed mice showed significant increases in GFAP intensity throughout the hippocampus compared to control chow-fed mice (Fig. 2A,B). The number of GFAP+ astrocyte cell bodies was significantly increased in WD-fed B6 mice (Fig. 2C). Analysis of GFAP immunoreactivity (Fig. 2D,E) and GFAP+ cell bodies (Fig. 2F) in the entorhinal cortex of mice fed WD also showed significant increases compared to control chow-fed B6 mice. IBA1 immunoreactivity and IBA1+ cell number were also significantly increased in the hippocampus and entorhinal cortex in mice fed WD compared to the control diet (Fig. 3).

**Figure 4:**
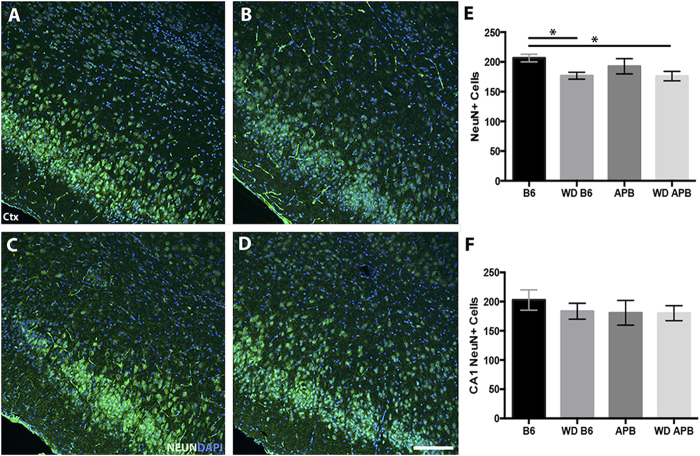
Small but significant changes in neuron number in response to diet in B6 or B6.*APB*^*Tg*^ mice. (**A–E)** The number of neurons (NeuN+, green) in the entorhinal cortex of B6 mice fed a western diet (**B**) show a significant decrease compared to mice fed a control diet (P = 0.0101) as well as a significant loss in B6.*APB*^*Tg*^ (**D**) compared to B6 control mice (**A**) (P = 0.0242, n = 5, **E**). No further decreases are seen in B6.*APB*^*Tg*^ fed either a western diet (**D**) or a control diet (**C**,**E**) (P = 0.4, n = 5). **(F)** Similarly, no significant difference was seen in the number of CA1 hippocampal neurons in B6 or B6.*APB*^*Tg*^ mice fed either a western diet or control diet (P = 0.74, n = 5). Scale bars: 160 μm.

Although there was no overt changes to cortical and hippocampal size, the number of neurons in the hippocampus and entorhinal cortex from B6 mice fed a western diet was determined. WD-fed B6 mice showed a small but significant decrease in NeuN+ neurons within the hippocampus compared to B6 mice fed the control diet (Fig. 4). A similar trend was observed in the entorhinal cortex although the decrease was not statistically significant. Collectively, these data showed that the major impact of the western diet was to induce glial responses (reactive astrocytosis and microglia/monocyte activation) in AD-susceptible brain regions and this correlated with significant but only subtle neuronal cell loss. Furthermore, the hippocampus region appeared more affected by diet-induced changes than the entorhinal cortex.

### The western diet exacerbates amyloid deposition, but not overt neuronal phenotypes in B6.*APB* ^*Tg*^ mice

**Figure 5:**
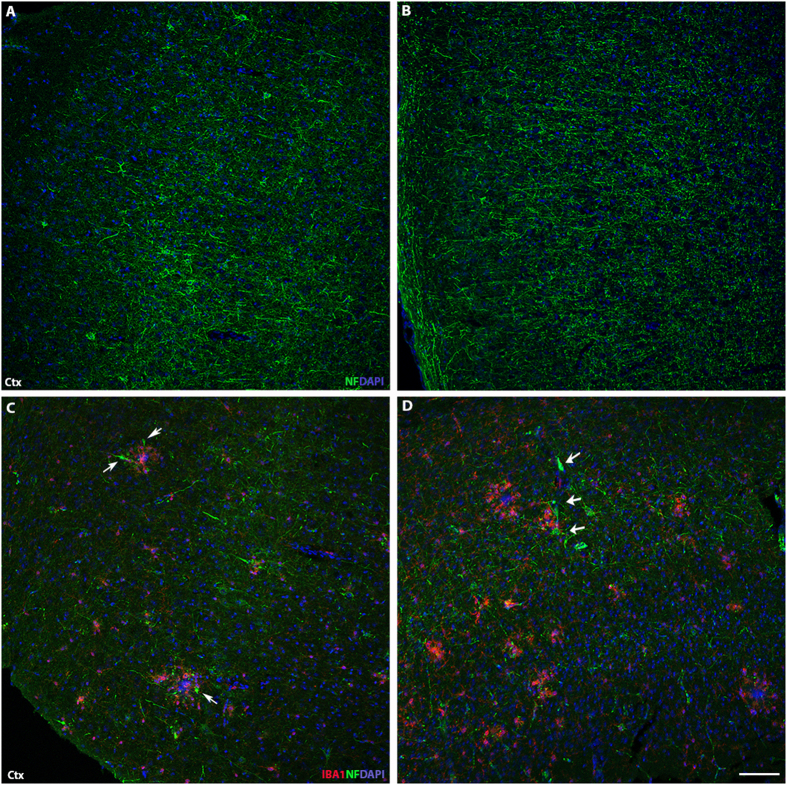
The western diet does not exacerbate dystrophic neurites in B6.*APB*^*Tg*^ mice. **(A,B)** Assessment of neurofilament (NF, green) shows no significant accumulation of dystrophic neurites (swollen segments of axons, arrows) in either B6 mice fed a control (**A**) or western diet (**B**). (**C,D**) The number of dystrophic neurites surrounding plaques (shown here by IBA1+ cell clusters, red) were not significantly different between diets. Fewer than 5 dystrophic neurites were observed around each of at least six plaques per section per six B6.*APB*^*Tg*^ mice fed a western diet or control chow. Scale bars: 160 μm.

Next, the impact of the western diet on AD progression was assessed using B6.*APB*^*Tg*^ mice, a commonly used mouse model for AD (see methods). They show plaque deposition from 4–6 mos that increases until approximately 10 mos^40^,^41^. However, in contrast to human AD, B6*.APB*^*Tg*^ mice show little to no neuronal cell loss, even at older ages^42^,^43^. B6.*APB*^*Tg*^ mice were fed a western diet prior to and throughout plaque deposition (from 2–10 mos). Despite the increase in glial activity due to the long-term consumption of the western diet (Figs 2 and 3), there was no significant increase in neuronal cells loss in the hippocampus and only a small but significant decrease in entorhinal cortical neurons in B6.*APB*^*Tg*^ mice fed a western diet compared to a control diet (Fig. 4). To further analyze the affects of the western diet on neurons, an assessment of axons was done. Previous reports have identified axonal swellings (dystrophic neurites) accumulating around Aβ deposits in the B6.*APB*^*Tg*^^44^,^45^. The western diet did not induce any noticeable dystrophic neurites in B6 mice (Fig. 5A,B). Dystropic neurites were associated around Aβ plaques but no significant difference was seen between mice fed a western diet or the control chow (Fig. 5C,D).

**Figure 6:**
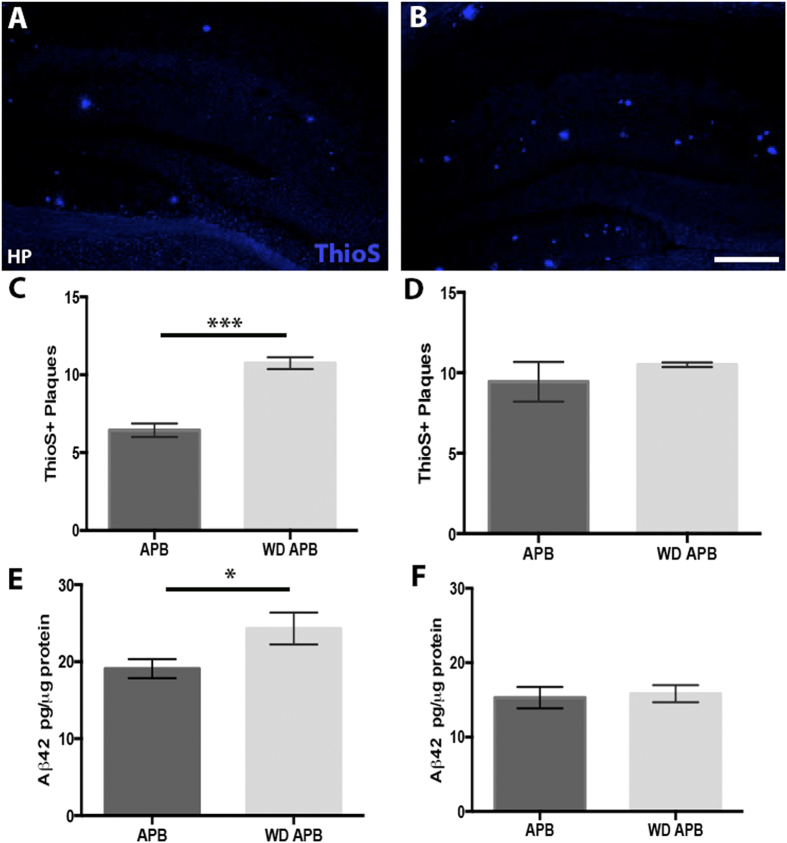
Hippocampal amyloid-beta plaque burden significantly increases in B6.*APB*^*Tg*^ mice fed the western diet. (**A**–**C**) Assessment of plaque number using Thioflavin S (ThioS, blue) of the hippocampus showed a significant increase in plaque number in the hippocampus of B6.*APB*^*Tg*^ mice fed a western diet **(B)** compared to a control diet (**A**) (p = 0.0008, n = 4, **C**). (**D**) Interestingly, this increase was not observed in the cortex (p = 0.5, n = 4). (**E**,**F**) The region specific increase in plaque burden was confirmed by measuring Aβ42 levels (by ELISA) that showed a significant increase in Aβ42 levels in the hippocampus (**E**, P = 0.464, n = 6), but not the cortex (**F**, P = 0.78, n = 6) of B6.*APB*^*Tg*^ mice fed the western diet (**E**) compared to the control diet (**F**). Scale bars: 160 μm.

Given glial responses can have both positive and negative impacts on amyloidosis^29^,^46^,^47^, the impact of the western diet on plaque deposition in B6.*APB*^*Tg*^ was assessed using quantification of ThioS labeled plaques and Aβ42 levels by ELISA. A significant increase in ThioS labeled plaque number and size was observed in the hippocampus but not the entorhinal cortex, (Fig. 6) and this was supported by the ELISA data that showed a significant increase in and Aβ42 levels in the hippocampus. This increase of amyloid plaques and Aβ42 levels in the hippocampus is consistent with the hippocampus being more susceptible to neuroinflammatory responses induced by the western diet compared to the cortex (Figs. 2 and 3). This data suggests that at least some of the glial responses induced by the western diet are potentially damaging.

### The western diet increases microglia/monocyte responses in B6 and B6.*APB* ^*Tg*^ mice

**Figure 7:**
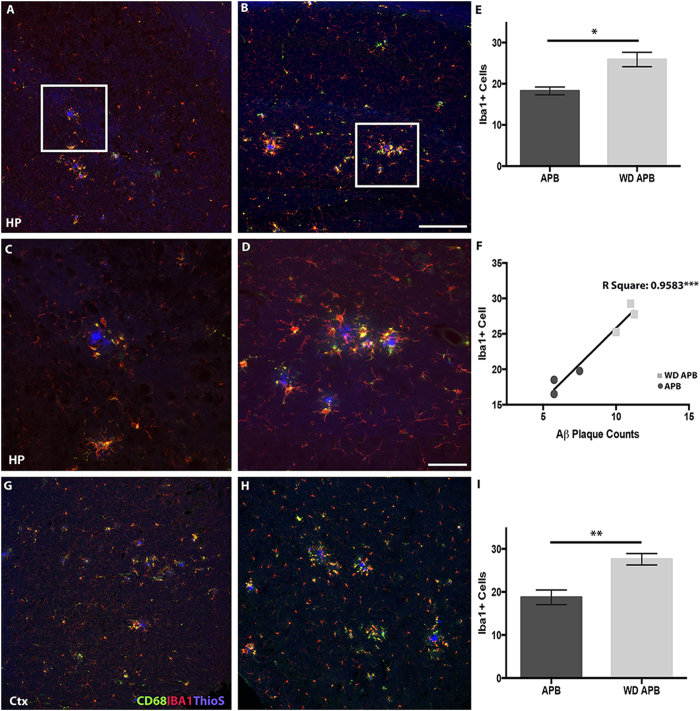
The western diet increases phagocytic microglia/monocyte around plaques in B6.*APB*^*Tg*^ mice. (**A**–**E**) Microglia/monocytes (Iba1, red) expressing CD68 (green) are significantly increased in regions of plaques in the hippocampus (blue, ThioS) in B6.*APB*^*Tg*^ mice fed the western diet **(B,E,D)** compared to the control diet (**A,C**) (p = 0.0184, n = 4, **E**). Middle panels (**C,D**) show high-resolution images from the white boxes seen in top panels (**A**,**B**). Although images were captured using exactly the same parameters, there appears to be higher levels of background ThioS staining in tissue from WD mice (**B**,**D**) compared to control diet (**A**,**C**). (**F**) There is a strong correlation between the number of IBA1+ cells and amyloid-β plaque number in the hippocampus (p = 0.0007, R^2^ =  0.9583, n = 3). **(G**–**I)** Microglia/monocytes are significantly increased in the entorhinal cortex surrounding plaque in western diet-fed B6.*APB*^*Tg*^ mice (p = 0.0086, n = 4). Blue = DAPI. Scale bars: A,B,G,H = 160 μm; C,D = 40 μm.

Microglia/monocytes become activated in response to Aβ deposition^29^,^32^,^33^. Therefore, to further assess microglia/monocyte responses around plaques we quantified IBA1, CD68 (a marker of phagocytosing cells) and TREM2 (a possible early driver of neuroinflammation). B6.*APB*^*Tg*^ mice fed the western diet showed a significant increase in the number of IBA1+ cells around plaques in hippocampus (Fig. 7A–D). The majority of these cells also expressed CD68, a marker of activated or phagocytic cells (Fig. 7A–D). Interestingly, there was also a significant increase in IBA1+ cells surrounding plaques in the entorhinal cortex of mice fed the western diet (Fig. 7E–H) despite the fact that the overall levels of plaque burden in the cortex was not increased.

**Figure 8:**
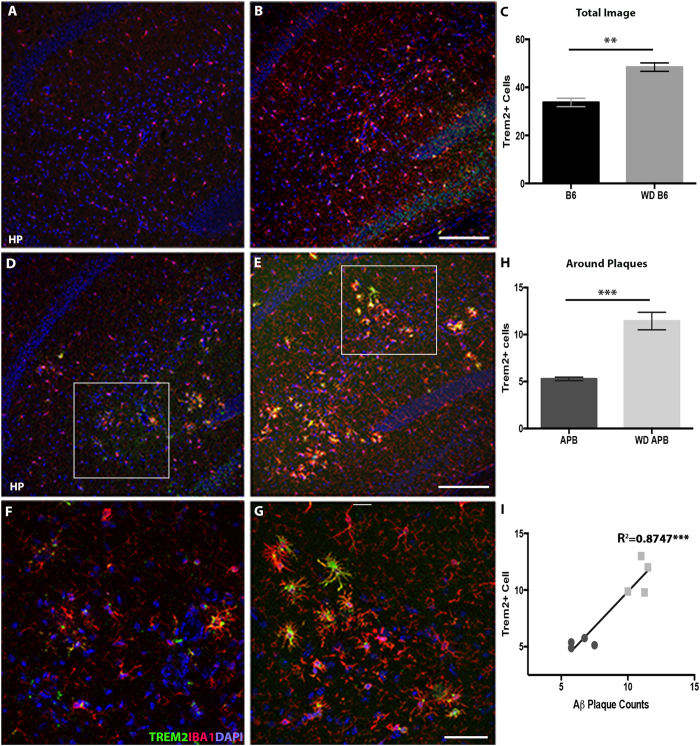
TREM2+ IBA1+ microglia/monocytes increase after chronic consumption of a western diet in both B6 and B6.*APB*^*Tg*^ mice. (**A**–**C**) TREM2+ (green) microglia/monocytes (IBA1+, red) increase within the hippocampus of B6 mice fed the western diet (**B**) compared to the control died (**A**). The total number TREM2 expressing IBA1+ cells are significantly increased (p = 0.001, n = 4, **C**). **(D**–**G)** TREM2+ IBA1+ cells also significantly increase within the hippocampus of B6.*APB*^*Tg*^ mice fed the western diet (**B**,**D**) compared to the control died (**A**,**C**) (p = 0.0006, n = 4, **H**). **(I)** There is a strong correlation between the number of TREM2+ IBA1+ microglia/monocytes and the number of amyloid-β plaques in the hippocampus (p = 0.0035, R^2^ = 0.8432, n = 4). Blue = DAPI. Scale bars: A,B = 160 μm, D,E = 160 μm, F,G = 40 μm.

Since *TREM2* is an important modulator of immune responses including phagocytosis, and recent studies have identified an important role for TREM2 in AD susceptibility and progression^30^,^32^,^48^, we assessed the number of TREM2+ cells in B6 and B6.*APB*^*Tg*^ mice fed the western diet compared to control diet. TREM2+ cells increased in the hippocampus of B6 mice (Fig. 8A–C), showing that chronic consumption of a western diet is sufficient to induce TREM2+ microglia/monocytes even in the absence of the APP/PS1 transgenes. As expected, TREM2+ cells were also increased in B6.*APB*^*Tg*^ mice fed the western diet compared to mice fed control diet (Fig. 8D–H). Given that TREM2+ cells have been shown to exacerbate plaque deposition in AD models^29^,^33^, we specifically counted the number of TREM2+ cells surrounding plaques in B6.*APB*^*Tg*^ mice. There was a significant increase in the number of TREM2+ cells surrounding plaques in the hippocampus (Fig. 8H) of B6.*APB*^*Tg*^ mice fed the western diet compared to B6.*APB*^*Tg*^ mice fed the control chow. Furthermore, there is a strong correlation (r^2^ = 0.8747) between TREM2+ cells and plaque number in B6.*APB*^*Tg*^ mice fed the western diet (Fig. 8I).

## Discussion

The increase in many diseases in western societies, including cardiovascular disease, type II diabetes, certain cancers and Alzheimer’s disease^38^,^49^, can be linked to dietary changes although little is known about how diet contributes to disease susceptibility and progression. One of the challenges has been that changes in dietary consumption over time have not been simple, but the result of changes in multiple dietary factors. Although some studies have modeled one or two of these dietary changes^17^,^18^,^19^,^20^,^21^, the western diet developed for this study is the first to incorporate the majority of the dietary components that make up the average diet in westernized countries. These components include being higher in animal fat and animal protein, lower in essential nutrients and higher in simple carbohydrates including high fructose corn syrup. We assessed the impact of this diet on AD susceptibility using B6 mice, and on AD progression using B6.*APB*^*Tg*^ mice. Long-term consumption of the western diet induced glial responses in the brain and exacerbated plaque load, particularly in the hippocampus. Future studies, assessing specific components of the diet, will enable us to determine whether multiple or specific aspects of the western diet are more critical for glial cell activation.

Current figures suggest one in three older adults in the USA are considered obese^50^. B6 mice fed the western diet for 8 months became obese (but not diabetic). These results suggest that our study could be an ideal model for mid-life obesity, a key risk factor for AD. A recent study shows that mid-life obesity increases risk of AD by more than 7% and in combination with physical inactivity, poor lifestyle choices could account for more than 25% of AD cases^51^. Interestingly, the western diet did not significantly increase weight in DBA/2J (data not shown), confirming that genetic factors play a role in diet-induced obesity^52^,^53^. It is not clear why a western diet and/or diet-induced obesity increase the risk for AD. However, multiple studies have linked saturated fats and simple carbohydrates with an increased risk for AD^54^,^55^, dietary components that were both incorporated into the western diet developed in this study. These dietary deficiencies have been reported to trigger inflammatory responses in peripheral tissues and the brain^56^,^57^,^58^. Therefore, individual ingredients within the diet or a combination of the ingredients, independent of obesity itself, could be contributing factors leading to increased innate immune responses and increased plaque load in the brain. This possibility is supported by a recent study that shows lower weight individuals have an increased risk of AD^10^.

In our study, mice fed the western diet showed a significant increase in microglia/monocyte activation. However, it is not clear whether this increase is specific to resident microglia or whether monocytes (or macrophages) enter the brain in response to, for instances, changes in cytokines or chemokines or if it is due to a breakdown of the blood brain barrier. In severe cases of neurodegeneration, monocytes infiltrate into the central nervous system^29^,^59^,^60^. One study showed that chronic expression of IL-1β, a proinflammatory cytokine, induced leukocyte infiltration independent of blood-brain barrier breakdown or neurodegeneration^61^. Further work is required to determine whether aspects of the microglia/monocyte response are beneficial, detrimental or both. As we age, there is a general shift from anti-inflammatory to pro-inflammatory cytokines in the brain, which has been associated with cognitive decline^62^ and this could be exacerbated by diet or other environmental factors. However, a recent study showed that AD mice deficient in IL10 showed a decrease in microglia activation and plaque burden suggesting that in some circumstances, some anti-inflammatory responses are not beneficial^46^.

Chronic consumption of the western diet-fed B6 and B6.*APB*^*Tg*^ mice caused a significant increase in TREM2 expressing microglia/monocytes. TREM2 has been implicated in a number of age-related neurodegenerative diseases including AD^30^,^31^ and frontotemporal dementia^63^, suggesting it may play a common age-specific role. A recent study showed that TREM2 deficient AD mice showed significantly decreased numbers of inflammatory macrophages, infiltrating monocytes and a reduction in plaque load^29^. Further, AD mice heterozygous for *Trem2* show a decreased number of plaque-associated microglia suggesting that TREM2 plays a key role in the recruitment of macrophages to areas of injury in the brain^64^. Collectively, these studies suggest TREM2 plays an important role in regulating microglia/monocyte activation in AD but its exact role is not clear. Our study is the first to show an increase in TREM2+ cells in response to a western diet suggesting TREM2 could be a critical molecule in modulating microglia/monocyte activation in response to dietary factors as well as in neurodegenerative diseases. Further, we show a strong correlation between TREM2+ microglia/monocytes and increased hippocampal plaque load in AD mice. Given that dietary factors alone caused an increase in TREM2+ cells, treatments that target TREM2 may be a viable option for obesity- or diet-induced cognitive decline.

## Additional Information

**How to cite this article**: Graham, L. C. *et al.* Chronic consumption of a western diet induces robust glial activation in aging mice and in a mouse model of Alzheimer’s disease. *Sci. Rep.* **6**, 21568; doi: 10.1038/srep21568 (2016).

## Figures and Tables

**Table 1:** The western diet (TestDiet 5W80) mimics the diet of western societies.

	Western Diet	Control Diet
**Total Protein**	16.0%*	19.0%**
**Total Fat**	16.4%*	4.6%**
Cholesterol	1,739 ppm	240 ppm
Linoleic/Linolenic Acids	1.46%	2.3%
Omega-3 Fatty Acids	0.22%	0.33%
Total Saturated Fatty Acids	8.04%	1.01%
Total Monounsaturated Fatty Acids	4.5%	1.04%
**Total Fiber**	4.3%	4.2%
**Total Carbohydrates**	57.6% (including high fructose corn syrup and sucrose that is absent in control diet)	65.4%
**Total Energy (kcal/g)**^2^	4.43%	4.02%

^*^Obtained from animal-based products.

^**^Obtained from plant-based products.
